# Direct, non-medical out-of-pocket expenditures for mothers of moderate or late preterm infants in a level II NICU: Comparison of Alberta Family Integrated Care versus standard care

**DOI:** 10.1016/j.pecinn.2024.100365

**Published:** 2024-12-20

**Authors:** Jacqueline M. Wilson, Oyinda Obigbesan, Elena Lopatina, Karen M. Benzies

**Affiliations:** a Faculty of Nursing, University of Calgary, PF3280C, 2500 University Drive, NW Calgary, AB T2N 1N4, Canada; b Cumming School of Medicine, University of Calgary, PF3280C, 2500 University Drive, NW Calgary, AB T2N 1N4, Canada; c Department of Community Health Sciences, Cumming School of Medicine, University of Calgary, Alberta Virtual Pain Program, Alberta Health Services, 2500 University Drive, NW Calgary, AB T2N 1N4, Canada; d Faculty of Nursing, University of Calgary, PF3280C, 2500 University Drive, NW Calgary, AB T2N 1N4, Canada

**Keywords:** Family-integrated care, Preterm infant, Neonatal care, Financial burden, Family-centered care, Parental involvement

## Abstract

**Objective:**

To compare direct, non-medical out-of-pocket expenditures (OOPE) between mothers receiving Alberta Family Integrated Care (FICare™) versus standard care (SC) during their infant's neonatal intensive care unit (NICU) admission and explore factors influencing spending extremes.

**Methods:**

In this exploratory, concurrent mixed-methods sub-study, we compared mother-reported OOPE from Alberta FICare™ and SC parent journals. We thematically analyzed hand-written notes from 30 journals with the highest and lowest 5 % of OOPE.

**Results:**

There was no difference in total direct, non-medical OOPE between Alberta FICare™ (*n* = 194) and SC (*n* = 132) groups (*U* = 12,679.50, *p* = 0.882). Compared to mothers receiving SC, mothers receiving Alberta FICare™ reported spending less for parking (*U* = 970.00, *p* < 0.001) and more for food (*U* = 14,857.50, *p =* 0.014) and lodging (*U* = 15,160.00, *p* < 0.001). Spending extremes related to travel and proximity of the NICU to their home.

**Conclusion:**

Total family financial burden was similar between groups; there were differences in spending categories. Supports to offset OOPE, particularly for families living distant to the NICU or facing transportation challenges, would reduce financial burden and could enhance family-integrated care.

**Innovation:**

This novel analysis describes mother-reported OOPEs and strategies to mitigate financial barriers to family integrated care.

## 1 Introduction

In Canada and Alberta, preterm birth rates are 8.4 % and 9.4 %, respectively [1]. Of preterm infants, approximately 85 % are born moderate (32^0/7^ to 34^6/7^ weeks gestation) or late (35^0/7^ to 36^6/7^ weeks gestation) preterm [2]. Facing high risk of adverse outcomes, mortality, and morbidity, preterm infants are often admitted to the neonatal intensive care unit (NICU) to receive comprehensive care with advanced technology from specialized staff who are experienced in caring for critically ill newborns [3].

Globally, high health care costs have been attributed to preterm birth and admission to the NICU [2,[4], [5], [6]]. In Canada, the median cost of inpatient NICU hospitalization for a preterm infant born between 33 and 36 weeks gestation is $11,810 CAD (IQR $6410–$19,800), demonstrating considerable economic impact on the Canadian health system [7]. These costs are even higher for infants born before 33 weeks, as there is an inverse relationship between an infant's gestational age and length of stay (LOS) in the NICU [2]. Comparatively, in 2021–2022, the average inpatient hospitalization cost estimated for term infants (>37 weeks gestation) in Canada ranged between $1212.00 CAD to $1822.00 CAD depending on the mode of delivery and whether the infant was a singleton or multiple birth [8].

While the economic impact of preterm birth and NICU hospitalization on the health system is well-studied [2,[4], [5], [6],^9^,^10^], the out-of-pocket expenditures (OOPE) incurred by parents of preterm infants during their NICU hospitalization is under-reported in most studies [5,11,12]. A systematic review of 44 studies examining family's OOPE in high- and low-income countries estimated a financial burden of 5 % to 10 % of a family's annual income during NICU birth hospitalization; however, the evidence was deemed low quality across studies [5]. Most previous studies neglected to estimate the direct, non-medical OOPE incurred by families across categories including food, transportation, and accommodation [2,[13], [14], [15]]. For example, King and colleagues' [5] systematic review found only two of 44 studies reported direct, non-medical OOPE categories during NICU hospitalization, despite the multiple direct, non-medical OOPE that are necessary to maintain family life during the preterm infant's hospitalization [16].

The potential to incur a high financial burden during the infant's hospitalization poses as an additional stressor for families during an already overwhelming time. The experience of having an infant in the NICU is stressful and potentially traumatic for parents, which may have lasting implications on the parent-infant relationship [17,18]. To better support families during this stressful period, NICUs worldwide have shifted towards models of family-centered care to support early parent-infant relationships, enhance family outcomes, and empower family involvement in the provision of non-medical care (e.g., feeding, diapering, and skin-to-skin care) [19,20]. These family-centered care interventions have been associated with positive outcomes for infants and families, with a systematic review of randomized controlled trials reporting increased (a) infant weight gain, (b) maternal knowledge and skills, and (c) care satisfaction compared to standard care [21]. This review found no statistically significant group differences in NICU LOS or infant neurobehavioral development [21]. However, individual studies have demonstrated the potential for family-centered care interventions to reduce NICU LOS, which may subsequently reduce health system costs [9,[22], [23], [24]].

Despite the global shift towards implementing family-centered care interventions that require increased family involvement in the NICU, there is limited understanding of how family's direct, non-medical OOPE (e.g., travel, parking, food, and childcare for older siblings) may be affected. As family-centered care interventions often require greater family presence and involvement in the NICU, it is reasonable to expect that direct, non-medical OOPE may also increase. However, increasing family involvement in the NICU also has the potential to decrease the infant's overall NICU LOS. A cluster randomized controlled trial (cRCT) examining Alberta Family Integrated Care (FICare™), a family-centered care model of care, found that the intervention significantly reduced NICU LOS by 2.55 days [22]. As such, we could also expect that the direct, non-medical OOPE for families receiving Alberta FICare™ may also decrease.

Better understanding the factors influencing spending extremes could inform potential supports to facilitate the integration of families of moderate and late preterm infants in the NICU. Therefore, the objective of this study was to explore and compare mother-reported, direct, non-medical OOPE and its categories from infant admission to discharge between mothers who received Alberta FICare™ versus standard care (SC). In Phase 1 (quantitative), we asked: What is the effect of Alberta FICare™ on direct, non-medical OOPE for mothers of a moderate or late preterm infant during their NICU hospitalization? Given that Alberta FICare™ significantly reduced NICU LOS [22], we hypothesized that mothers in the Alberta FICare™ group would report significantly lower direct, non-medical OOPE between NICU admission and discharge compared to mothers in the SC group. In Phase 2 (qualitative), we asked: What factors influenced the highest and lowest direct, non-medical OOPE for both groups.

### 1.1 Conceptual framework

Hodek et al. [12] recommend evaluation of OOPE in four domains: (a) direct medical costs such as inpatient hospitalization, outpatient clinic visits, medication, and medical equipment; (b) direct, non-medical costs such as parking, meals, transportation, and childcare; (c) indirect costs including lost time, lost income, and missed work; and (d) intangible costs related to emotional health, social health, and quality of life. Given that (a) publicly funded health care insurance in Canada covers direct medical costs [25], (b) the national maternity benefits program covers 55 % of indirect costs related to lost time from work (up to a $668 weekly maximum) [26], and (c) our research questions did not include indirect and intangible cost estimates, we focused on direct, non-medical OOPE.

## 2 Methods

### 2.1 Study design and setting

As a sub-study of the Alberta FICare™ cRCT, we analyzed direct, non-medical OOPE using an exploratory, concurrent mixed methods design with quantitative (Phase 1) and qualitative (Phase 2) data analyzed separately and triangulated results in the discussion [27]. This evaluation followed an a priori analysis plan of the larger Alberta FICare™ cRCT, which was conducted between December 2015 and July 2018 across 10 level II NICUs (five Alberta FICare™ and five SC) in Alberta, Canada. See Benzies et al. [22] for details on the protocol for the larger Alberta FICare™ cRCT.

Alberta has a single, integrated system providing publicly funded health services to 4.5 million residents [28]. While definitions and the scope of care may vary by jurisdiction, Level II NICUs typically provide speciality care to infants who may be born prematurely, underweight, and/or moderately ill, but who are not expected to require interventions such as sustained life support or immediate care from pediatric subspecialists [3]. The University of Calgary, Conjoint Health Research Ethics Board (CHREB ID 15-0067), University of Alberta, Health Research Ethics Board (Pro00060324), and Covenant Health Research Ethics Board (ID 1762) approved this study.

### 2.2 Participants

We included mothers of preterm infant(s) born between 32^0/7^and 34^6/7^ weeks gestational age, with primary admission or transfer within 72 h of birth to a level II NICU. We capped infant gestational age at 34^6/7^ weeks to ensure families received a minimum 1-week exposure to Alberta FICare™ because otherwise healthy infants who are gaining weight are generally discharged from the NICU at approximately 36 weeks gestational age [29]. We excluded mothers with (a) health, social, or language challenges, (b) triplets or higher order multiple births, and (c) infants with severe congenital anomalies or requiring palliative care. At intervention sites, we included mothers who agreed to spend at least 6 h per day with their infant because this was deemed the minimum exposure to the intervention to achieve an effect.

### 2.3 Intervention: Alberta Family Integrated Care

Alberta FICare™ is a model of family-centered care, which was iteratively co-designed with parents and healthcare providers, that trains neonatal care providers to educate and support parents to become partners in their infant's NICU care team [22]. In Alberta FICare™ online learning modules, providers learn about (a) relational communications, (b) parent education, and (c) parent support. To facilitate involvement in care, mothers in the Alberta FICare™ group received a parking pass. Infants and mothers in the SC group received care as usual, which is underpinned by a philosophy of family-centered care [30] but does not include the specific training, strategies, and tools of Alberta FICare™.

### 2.4 Data collection

Mothers recorded OOPE in Canadian dollars (CAD) using an investigator-designed parent journal. See Supplementary File 1 for the data collection pages and definitions of OOPE provided to participants. Mothers self-reported their direct, non-medical OOPE and recorded hand-written notes about their NICU stay in the parent journal. Direct, non-medical OOPE categories included parking, food, lodging, childcare, household support, travel for family support, and miscellaneous items. Aligned with recommendations by Hodek et al. [12], we encouraged participants to record OOPE and hand-written notes daily.

Using an online investigator-designed questionnaire, we collected sociodemographic and health information for mothers and infants at admission and discharge. For mothers, we collected: relationship status (partnered or not partnered), education, employment, income, ethnicity, country of birth, primary language spoken at home, parity (number of pregnancies carried past 20^0/7^ weeks gestational age [31]), cRCT enrolling hospital, and singleton or twin birth. For infants we collected gestational age.

### 2.5 Procedures

Randomization and masking for the larger Alberta FICare™ cRCT are reported elsewhere [22]. After informed, written consent and within 72 h of infant admission, participants received a parent journal and completed the online sociodemographic questionnaire [32]. At discharge, a research nurse collected parent journals for secure transport to the research office. JMW (first author) scanned each parent journal, extracted data, and mailed the parent journal back, unless the mother declined. Parent journal and cRCT data were linked using study ID.

### 2.6 Data analysis

For each participant recording OOPE in the parent journal, the time horizon for data collection was from infant NICU admission to discharge. Data were collected over three years; as such, all OOPEs were adjusted for annual inflation and reported in 2024 Canadian dollars (see Supplementary File 2).

Prior to Phase 1 analysis, we examined data for out-of-range and missing values, and assumptions for statistical tests. We discussed cases with out-of-range values and excluded those with extreme OOPE (e.g., over $10,000 for family member to travel from another country to care for siblings), or OOPE unrelated to NICU hospitalization (e.g., annual automobile insurance).

**Fig. 1:**
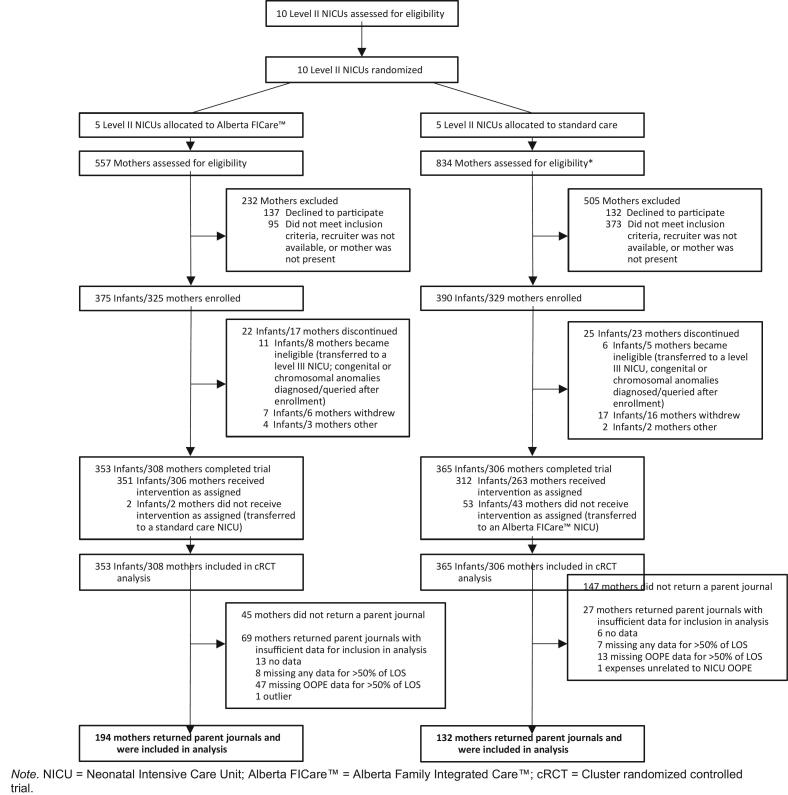
Consort flow diagram outlining inclusion of participants in the study. *Note.* NICU = Neonatal Intensive Care Unit; Alberta FICare™ = Alberta Family Integrated Care™; cRCT = Cluster randomized controlled trial.

Despite encouragement for participants to record OOPE daily, many days had missing values. We defined a day with data as (a) any dollar amount recorded, including zero, (b) any OOPE category crossed out to indicate no spending, or (c) “not applicable” recorded. If a case included twins with different LOS, the longest LOS was used to calculate the proportion of days with data. We assumed zero parking expenditure for mothers in the Alberta FICare™ group because they received a parking pass as part of the study. We included cases in the analyses if the proportion of days with OOPE data was greater than 50 % of the infant's LOS. We selected 50 % as because it enabled us to retain the maximum number of cases for analysis while limiting the number of cases with large amounts of potentially missing OOPE data. We did not deem it appropriate to replace missing OOPE values due to the wide variability in spending between mothers and between days in the NICU. Ultimately, 69 (22.4 %) cases were removed from the Alberta FICare™ group and 27 (8.8 %) cases were removed from the SC group because the 50 % cut-off for OOPE data was not met (Fig. 1). Supplementary File 3 includes the analysis of all cases and analysis of cases with any journal data for 50 % of the infants LOS. Although expectations about recording OOPE were the same between groups, we speculate that the group differences in missing data may be due to increased expectations for mothers in the Alberta FICare™ group to be involved in their infant's care and record additional information about their infant and their NICU experience in the parent journal.

Baseline characteristics were compared between groups using independent samples *t*-tests and Pearson χ^2^. Given that data were skewed for total and all categories of direct, non-medical OOPE, groups were compared using Mann Whitney U. IBM® SPSS, Version 28 [33] was used for analysis with alpha set at *p* < 0.05.

Findings from Phase 1 guided case selection for the Phase 2 qualitative analysis. Specifically, in Phase 2, hand-written notes in parent journals from mothers reporting OOPE in the highest (≥95th) and lowest (≤5th) percentiles were thematically analyzed following Braun and Clarke's six phases of thematic analysis [34]. This analysis included, (1) becoming familiar with the data by reading the journals and noting sections where OOPE categories were mentioned; (2) manually generating initial codes from the data; (3) collapsing the codes into broader themes; (4) reviewing the themes and additional recoding after rereading the data; (5) defining the themes and determining if subthemes were needed; and (6) writing the qualitative portion of the manuscript [34]. Results from Phases 1 and 2 were triangulated in the discussion.

## 3 Results

Of the 654 mothers of 765 infants who were enrolled in the Alberta FICare™ cRCT [22], 422 (64.5 %) mothers returned a parent journal. Of these, 326 parent journals were analyzed (Alberta FICare™ *n* = 194; SC *n* = 132) in Phase 1 (Fig. 1).

### 3.1 Maternal and infant characteristics

On average, mothers in the Alberta FICare™ group (*n* = 193) were 31 (*SD* = 5.28) years old; mothers in the SC group (*n* = 126) were 32 (*SD* = 5.08) years old, *t*(317) = 1.403, *p* = 0.161. Infants in the Alberta FICare™ group (n = 194) had a mean LOS of 18.05 (SD = 8.25) days; infants in the SC group (n = 132) had a mean LOS of 19.25 (SD = 7.77) days, t*(*324) = 1.317, *p* = 0.189. Except that the Alberta FICare™ group included significantly more mothers from regional than urban hospitals (*χ*2(1) = 7.87, *p* = 0.005) there were no other group differences at baseline (Table 1).

**Table 1:** Maternal and infant characteristics by group.

Maternal characteristics	Alberta FICare™ (*n* = 194)		Standard care (*n =* 132)		*p*
	*n*	Frequency (%)	*n*	Frequency (%)	
Partnered^a^	193	185 (95.9)	129	126 (97.7)	0.38
Education	192		129		0.63
High school diploma or less		28 (14.6)		15 (11.6)	
Certificate/diploma		53 (27.6)		33 (25.6)	
College/university degree		111 (57.8)		81 (62.8)	
Employment status	191		129		0.54
Employed (Full time or part time)		58 (30.4)		33 (25.6)	
Maternity leave^b^		88 (46.1)		70 (54.3)	
Not employed and not seeking employment		26 (13.6)		16 (12.4)	
Other^c^		19 (9.9)		10 (7.8)	
Annual family income	167		112		0.71
< $40,000		7 (4.2)		5 (4.5)	
$40,000 to $79,999		28 (16.8)		23 (20.5)	
> $80,0000		132 (79.0)		84 (75.0)	
Ethnicity	192		128		0.30
Asian (South, East, Southeast)		18 (9.4)		20 (15.6)	
Black		6 (3.1)		6 (4.7)	
Other^d^		18 (9.4)		12 (9.4)	
White		150 (78.1)		90 (70.3)	
Born in Canada	192	156 (81.3)	128	92 (71.9)	0.05
English as primary language	192	179 (93.2)	128	111 (86.7)	0.05
Primiparous	193	109 (56.5)	131	67 (51.1)	0.34
Enrolled at urban hospital	194	148 (76.3)	132	117 (88.6)	0.005

Infant characteristics					
Singleton	194	164 (84.5)	132	104 (78.8)	0.18
Gestational age (weeks)	194		132		0.27
32 weeks		38 (19.6)		17 (12.9)	
33 weeks		53 (27.3)		37 (28.0)	
34 weeks		103 (53.1)		78 (59.1)	

*Note*. Sample sizes vary due to missing values. ^a^Partnered includes married, common-law, or live-in partner. Not partnered includes single or separated. ^b^ Mother had formalized Canadian maternal leave benefits at the time of demographic survey completion. ^c^Other includes student, not employed but seeking employment, disability or medical leave, and other (e.g. illness, unpaid leave, laid off, contract employment, self-employed). ^d^Includes Indigenous (e.g., First Nations, Inuit, Metis), Latin American, Middle Eastern, and Other/Mixed.

### 3.2 Phase 1: Quantitative

For total direct, non-medical OOPE, there was no significant difference between Alberta FICare™ and SC groups (*U* = 12,679.50, *p* = 0.882). See Table 2. There were significant group differences in OOPE categories for parking, lodging, and food. Compared to mothers in the Alberta FICare™ group, mothers in the SC group reported significantly higher parking expenditures, which was expected. Compared to mothers in the SC group, mothers in the Alberta FICare™ group reported significantly higher food and lodging expenditures, which was not expected. Mothers in both groups reported similar items under miscellaneous expenditures, including infant feeding supplies (e.g., breast pump, bottles), transportation (e.g., taxi fare, flight home), groceries (e.g., extra groceries for NICU, grocery delivery), infant items (e.g., clothes, car seat), postpartum items (e.g., breast pads, medications/supplements), and items to facilitate time in the NICU (e.g., phone data plan, pet boarding, toiletries).

**Table 2:** Comparison of direct, non-medical out-of-pocket expenditures between Alberta FICare™ and standard care groups.

Expenditure	Alberta FICare™ (*n* = 194)		Standard care (*n* = 132)		*U*	*p*
	Median (IQR)	Minimum, Maximum	Median (IQR)	Minimum, Maximum		
Total	692 (1446)	0, 8188	773 (1069)	25, 6556	12,679.50	0.882
Parking^a^	0 (0)	0, 0	119 (88)	0, 515	970.00	<0.001
Food	291 (478)	0, 3053	235 (275)	0, 2367	14,857.50	0.014
Lodging	0 (150)	0, 3121	0 (0)	0, 677	15,160.00	<0.001
Childcare^b^	0 (0)	0, 2494	0 (0)	0, 1485	2766.00	0.603
Household Support	0 (0)	0, 1026	0 (0)	0, 1378	12,956.50	0.740
Family Travel	0 (263)	0, 5012	0 (200)	0, 4552	13,890.50	0.148
Miscellaneous	20 (178)	0, 3590	12 (189)	0, 1895	12,914.50	0.889

*Note.* Expenditure values reported in 2024 Canadian dollars (CAD) and rounded to the nearest whole number.

IQR = Interquartile range (IQR = Q3-Q1).

a Mothers in the Alberta FICare™ group were assumed to have zero parking costs as they received a parking pass.

b Childcare compared only for multiparous mothers (Alberta FICare™, *n* = 84; standard care, *n* = 64).

### 3.3 Phase 2: Qualitative

Qualitative analysis included 15 cases with total direct, non-medical OOPE ≥95th percentile (Alberta FICare™ *n* = 9; SC *n* = 6), and 15 cases with total direct, non-medical OOPE ≤5th percentile (Alberta FICare™ *n* = 9; SC *n* = 6). Two themes were identified in relation to the spending extremes: (a) getting to the NICU and (b) proximity to the NICU. Verbatim quotes are attributed by study ID number, study group, and highest vs. lowest expenditure percentile.

#### 3.3.1 Getting to the NICU

Following discharge from hospital, maternal involvement in the NICU required transportation and parking. Transportation to the NICU was especially challenging for mothers recovering from caesarean birth who were advised not to drive. One mother noted, “[Doctors name]: no ‘being in a car’ 6 weeks but knows not reasonable. Check with insurance about driving” (ID 233, Alberta FICare™, highest). The following day, this mother noted, “Moved to a hotel today to be closer to baby”, which enabled the mother to walk to the NICU. Another mother with a caesarean birth noted the high cost of taxis when the family car was unavailable.

Only if I could get to spend more time but unfortunately we only have one car and her father had used it for work and for me to taxi everyday would cost us a lot and I can't afford that amount. On taxi alone one way is $40 so the little time I have to spend with her I make it very useful.

(ID 719, Alberta FICare™, lowest)

Some mothers had to coordinate transportation to the NICU around the schedules of others. Reliance on others for transportation and an inability to be in the NICU created guilt. One mother who had a caesarean birth noted, “Only one visit today as Grandma was driving mom around to do errands today. Feel bad asking for a ride back to the hospital. Feeling guilty for not visiting more” (ID 288, SC, lowest).

Mothers also remarked spending more on transportation and parking to facilitate care of older siblings:

Mom and dad traveled to hospital separately. Extra gas as we live half an hour from hospital. Toddler did not have care so dad only able to stay for a few hours. As a mom I would love to stay in the NICU longer with my baby but also have to help at home (husband works shift work) and spend time with my toddler as well.

(ID 199, SC, highest)

Mothers wrote that parking was expensive and a stressor. After staying on the antepartum unit and prior to study enrollment, one mother noted, “Parking is always so expensive and disheartening to pay. Always a stress.” (ID 721, Alberta FICare™, highest). One mother in SC group (ID 288, lowest) received a parking pass from the NICU, possibly due to financial need.

#### 3.3.2 Proximity to the NICU

Across both groups, mothers who lived near the NICU reported lower expenditures for food, accommodation, and transportation to the NICU. One mother stated, “Because we live so close, we really don't have any extra expenses being in the NICU” (ID 254, Alberta FICare™, lowest). Even with active steps to reduce expenditures, such as receiving transportation from support people, borrowing instead of renting a car, staying with friends, and applying for federal government tax exemptions [35], mothers in both groups living further from the NICU generally incurred higher expenditures. One mother wrote:

According to the doctors we still have probably another week. It is getting very discouraging and sad but it's all we can do. It is getting very costly, as we are paying $100/night to stay here. That alone is stress enough. I hope we can soon go home.

(ID 622, Alberta FICare™, highest)

Mothers who required an ambulance or medical flight from a different jurisdiction had particularly high OOPE. The unexpected early birth necessitated purchase of clothes, toiletries, and infant care items that mothers would have normally brought from home. One mother from out-of-province (ID 647, Alberta FICare™, highest) spent over $6500 CAD on food, lodging, rental cars, family members flights, infant items such as a stroller to fly home, and other daily essentials that they did not have time to pack prior to the birth. In addition to the financial burden of being admitted far from home, this mother acknowledged the logistical burden of coordinating family travel to return home after discharge.

## 4 Discussion and conclusion

### 4.1 Discussion

In this exploratory, concurrent mixed methods sub-study in 10 Canadian level II NICUs, we compared mother-reported, direct, non-medical OOPE from infant admission to discharge between participants who received Alberta FICare™ versus SC. We hypothesized that mothers in the Alberta FICare™ group would report significantly lower *total* direct, non-medical OOPE compared to mothers in the SC group because they received a parking pass and had a shorter infant LOS [22]. While the median value for total direct, non-medical OOPE was lower for mothers in the Alberta FICare™ group than the SC group, the difference was not statistically significant; our hypothesis was not supported. If mothers in the Alberta FICare™ group had not received a free parking pass, it is likely that their total direct, non-medical OOPE would have been higher than mothers in the SC group.

In analyses of categories of direct, non-medical OOPE, consistent with our hypothesis, mothers in Alberta FICare™ group spent significantly less on parking compared to mothers in the SC group. However, inconsistent with our hypothesis, mothers in Alberta FICare™ group spent significantly more on food and lodging than mothers in the SC group. The higher food and lodging costs incurred by mothers in the Alberta FICare™ group may be associated with the group's increased presence in the NICU with their infant. Although mothers in Alberta FICare™ group agreed to be present in the NICU at least 6 h per day, they spent an average of 9 h in the NICU while mothers in the SC group spent significantly less time in NICU at 7.8 h per day [22].

Similar to our findings, families present in the NICU with their infant reported the highest spending in categories of food at the hospital, lodging near the hospital, and transportation in an Australian study of infants born at <34 weeks gestation [13]. These families reported wanting to bring food from home but often resorted to purchasing food due to minimal safe food storage options and the stress associated with food preparation in the NICU [13]. Parents interviewed by Lasiuk et al. [36] in a large Canadian city also described how their stress levels in the NICU and ability to cope were impacted by expenses such as transportation and hospital parking, food costs at the hospital, and childcare for other children. The financial stressors associated with the NICU were found to be particularly impactful on families who were self-employed, whose infant had an extended LOS, and who lived a far distance from the NICU [36].

In our study, the burden of travel to-and-from the NICU may also have affected mothers' spending decisions, as higher food and lodging costs were reported within the Alberta FICare™ group where there was a higher number of infants admitted to regional NICUs. The influence of mothers' proximity to the NICU on their stress levels, financial burden, and ability to be present with their infant in the NICU has been previously reported in qualitative studies examining mothers' experiences with the NICU environment [36,37]. It is noteworthy that regardless of study group, mothers who were from outside Alberta and whose infants were admitted to an Albertan NICU reported amongst the highest total direct OOPE, further demonstrating the potential influence of NICU proximity on families' financial burden.

Participants whose parent journals were analyzed in the qualitative phase of this study described their experience with OOPE in the parent journal, despite no specific prompt to comment on OOPE. Mothers acknowledged that OOPE kept them from being in the NICU as much as they wished, which contributed to stress, guilt, and embarrassment to ask about financial resources that could decrease their stress; these feelings are consistent with previous qualitative findings [38,39]. Almost half of parents (*N* = 27) interviewed by Miller et al. [40] received some external support to address their OOPE, such as parking vouchers, food cards, or hospital subsidized accommodations. Despite their access to these resources, the parents still reported feeling overwhelmed and underprepared for the costs associated with the NICU and their infant's treatment [40]. Over half of the parents interviewed described their desire to discuss the current and future costs associated with their infant's care, but only one parent did [40]. The potential hesitancy and guilt surrounding supports for OOPE that was reported in the parent journals highlights the potential utility of financial needs assessments for families in the NICU and the importance of ensuring NICU staff are aware of the financial resources available to NICU families at their local site [16].

#### 4.1.1 Limitations

Results of this study must be interpreted considering the limitations. Collecting daily mother-reported OOPE has the potential to reduce participant recall bias and aligns with the methodologic recommendations by Hodek et al. [12]. However, some mothers did not record OOPE for each day in the NICU, which resulted in missing data. To examine the effect of excluding cases with missing data, we reanalyzed the data using all cases. When all cases were analyzed, there were still no group differences for total direct, non-medical OOPE. Group differences continued for parking (mothers in SC spent more) and for lodging (mothers in Alberta FICare™ spent more); however, there was no longer a group differences for food. Thus, group differences in expenditures for food must be treated with caution. Furthermore, we did not require receipts to verify mother-reported expenses. In future studies, daily SMS reminders and utilizing checkboxes with spending ranges, rather than open-ended responses, may improve data quality.

Second, participants were primarily white, well-educated, and affluent, which limits generalizability to other populations. While we consented mothers to self-report data in the parent journal, we cannot be certain that other family members or support persons did not report data in the journal. In addition, we did not assess the effect of material hardship on mothers' abilities to be involved in the NICU. Given material hardship is independently associated with worse outcomes and greater health care utilization for low birthweight preterm infants in the United States [41], future studies should evaluate enhanced supports to reduce social inequities and stressors associated with involving families in care.

Finally, results related to parking and childcare expenditures should be treated with caution. Parking expenditures were assumed zero for participants in the Alberta FICare™ group because participants received a parking pass as part of the intervention, but families may have incurred additional parking costs that were not part of this analysis. Childcare expenditures were estimated only for mothers who reported being multiparous, which may have excluded primiparous mothers who incurred childcare expenditures for stepchildren.

#### 4.1.2 Implications for practice and recommendations

As healthcare systems shift towards family-centered care models with expectations for family involvement in the NICU, the OOPE incurred by families must be considered [16,42]. To reduce the direct, non-medical OOPE that families may incur to be present in the NICU, our recommendations are consistent with a previous integrative review [43], parent focus group [39], and consensus conference [44]. These recommendations include (a) flexible policies that permit sibling visitation, (b) private space in the hospital for families to eat and rest, (c) affordable accommodations in-unit or close to the hospital, and d) financial support for transportation and food [39,43,44]. Moving forward, opportunities to reinvest the health system savings attributed to family-centered care models into supports to encourage family involvement in the NICU must be explored, particularly for those who (a) experience material hardship, (b) live long distances from the NICU, and (c) face transportation barriers, such as recovery following a caesarean delivery.

### 4.2 Innovation

Family-centered care models improve parental experiences [45], enhance health outcomes [21], reduce infant LOS [22,46], and subsequently save health system costs [9]; however, the findings from this study highlight the financial effect on families when family-centered care models are implemented in the NICU, notably for food and lodging costs. The direct, non-medical OOPE that we collected using mother-reported journal data in this study provide new understanding into the financial burden incurred by families to be present in the NICU with their moderate or late preterm infant. These results are valuable to inform the implementation of family-centered care models within NICUs. In addition, to facilitate the collection of robust data to further demonstrate the economic impact of NICU admission on families, we highlight potential strategies to improve mother-reported OOPE data collection for future NICU research.

Perhaps most important to fostering innovation to support families in the NICU, is our novel interpretation of quantitative direct, non-medical OOPE data within the context of qualitative data describing families' experiences with spending in the NICU. To our knowledge, this is the first time that mother-reported direct, non-medical OOPE data has been analyzed concurrently with the voiced experiences and concerns of mothers with both high and low expenditures during their initial NICU admission. Our analysis provides a more in-depth understanding into families' needs during this crucial time and has the potential to inform targeted innovation of interventions. Such interventions have the potential to address parent-identified concerns regarding the financial impact of an infant's NICU admission, facilitate parental involvement in the NICU, and support families and healthcare systems to benefit from family-centered care models.

### 4.3 Conclusion

Overall, we found no group difference in total direct, non-medical OOPE between mothers receiving Alberta FICare™ and mothers receiving SC. Yet, families receiving Alberta FICare™ incurred higher direct, non-medical OOPE, particularly on food and lodging, while present in the NICU to gain the knowledge, skills, and confidence to enable earlier discharge [22]. The spending extremes reported by participants were influenced by their proximity to the NICU and transportation challenges, such as parking, coordinating with support persons for transportation, and limitations imposed by caesarean section recovery.

The economic impact of NICU admissions on the health system is well studied [2,[4], [5], [6],^15^], with recent evidence indicating that family-centered care models in the NICU have the potential to reduce health system costs by reducing infant LOS [9,[22], [23], [24]]. However, the financial burden incurred by families caring for hospitalized children remains under-reported and largely invisible to policymakers [5,11,12,16]. As a result, well-intentioned health systems leaders may implement family-centered care models meant to support infants and families, without recognizing the financial burden that these models may transfer to families during an already stressful time. To offset the direct, non-medical OOPE incurred by families when present in the NICU, health systems leaders must explore how system-level cost avoidance associated with family-centered care models can be reinvested into targeted interventions that facilitate family presence in the NICU, particularly for families who live distant to the NICU or face transportation challenges.

## Funding

This work was funded by Alberta Innovates–Health Solutions, Partnership for Innovation in Health Services Research [grant number 201400399] with in-kind support from Alberta Health Services, Covenant Health, and Faculty of Nursing, University of Calgary. The funder of the study had no role in study design, data collection, data analysis, data interpretation, or writing of the report. The first author received scholarships from: Canadian Institutes of Health Research; Alberta Health Services Maternal Newborn Child and Youth Strategic Clinical Network; Alberta Strategy for Patient Oriented Research Graduate Studentships in Patient Oriented Research; Alberta Children's Hospital Research Institute; Alberta Registered Nurses Education Trust; Canadian Nurses Foundation; and University of Calgary. The second author received scholarships from: the Alberta Children's Hospital Research Institute and the University of Calgary. The third author is supported by the Health System Impact Embedded Early Career Researcher Awards from the Canadian Institutes of Health Research [Funding Reference Number: 191646]. These funders had no role in study design, data collection, data analysis, data interpretation, or writing of the report.

## CRediT authorship contribution statement

**Jacqueline M. Wilson:** Writing – review & editing, Writing – original draft, Methodology, Formal analysis, Conceptualization. **Oyinda Obigbesan:** Writing – review & editing, Visualization, Formal analysis. **Elena Lopatina:** Formal analysis, Methodology, Writing – review & editing. **Karen M. Benzies:** Writing – review & editing, Writing – original draft, Supervision, Methodology, Conceptualization.

## Declaration of competing interest

Jacqueline M. Wilson, Oyinda Obigbesan, and Elena Lopatina declare no competing interests. Karen M. Benzies is the founder and CEO of Liminality Innovations Inc., a social enterprise that makes Alberta FICare™ accessible in jurisdictions outside Alberta.

## Data Availability

The data sets used and analyzed in the current study are available from the corresponding author on reasonable request.

## Appendix A Supplementary data

Parent Journal Example Image 1

Consumer Price Index adjustment factors used in the analysis Image 2

Additional analysis of direct non-medical out-of-pocket expenditures Image 3
