# Supplementary material for: Direct, non-medical out-of-pocket expenditures for mothers of moderate or late preterm infants in a level II NICU: Comparison of Alberta Family Integrated Care versus standard care
Source: PEC Innov. 2024 Dec 20;6:100365. doi: 10.1016/j.pecinn.2024.100365 (PMC11732068; doi:10.1016/j.pecinn.2024.100365)
Supplement: Supplementary file 1 — Parent Journal Example [file mmc1.pdf]

Supplementary File 1. Parent Journal Example

Investigator-designed parent journal used to capture direct, non-medical out-of-pocket expenditures and qualitative notes reported by mothers of infants in a level II Neonatal Intensive Care Unit.

PARENT and BABY INFORMATION

Mother's name:_________________________________

Father's name:_________________________________

Baby's name:__________________________________

Birth date:___________________________________

Birth weight:__________________________________

IMPORTANT PHONE NUMBERS

Parent distress lines:
Calgary [redacted]
Edmonton [redacted]

Health link: [redacted]
Emergency: [redacted]

If this journal is LOST, please contact me at:

phone: ______________________________________

e-mail: ______________________________________

Proof Copy: Not optimized for high quality printing or digital distribution

DEFINITIONS FOR DAILY EXPENSES

Travel: Mode of travel could be a personal vehicle, taxi, bus, etc. Write distance in kilometers from home/hotel to the hospital.

Parking: Cost of parking at or near the hospital.

Food: Cost of food purchased for mother and father during the time at the hospital. Please don't include cost of and groceries purchased for use at home or packed lunches.

Lodging: Extra costs of accommodation (hotel, Ronald McDonald House, bed and breakfast, etc.) during the time the baby is in the hospital. Please don't include costs if you are able to sleep at your own house.

Childcare: Extra costs for childcare during the time the baby is in the hospital. Please don't include regularly scheduled daycare or preschool program costs.

Time off from work: Paid leave includes vacation days and sick days - check "paid box". If time off work is unpaid, check "unpaid box" and estimate how much it costs you for the day.

Household support: This includes paying out of pocket for house cleaning, cooking, laundry, etc. during the time the baby is in the hospital.

Travel for family support: This includes the cost of a flight or other travel expenses for a family member (e.g. Grandmother, Sister) or friend to provide household support and childcare.

Miscellaneous: Cost of other items not listed above (e.g. renting a breast pump).

Proof Copy: Not optimized for high quality printing or digital distribution

Day 1 Information

Arrival time in NICU: __________ Departure time: ___________

Arrival time in NICU: __________ Departure time: ___________

Travel to hospital:

Mode: __________________________KM______________________

Travel from hospital:

Mode:___________________________KM______________________

Parking: $____

Food:$____

Lodging:$____

Childcare:$____

Time off work: [] paid [] unpaid

If unpaid, what is it costing you today?:$ ___________________

Household support:$_______________________________________

Travel for family support: $_______________________________

Miscellaneous: $____

Notes:____

____

Proof Copy: Not optimized for high quality printing or digital distribution
