# Supplementary material for: Direct, non-medical out-of-pocket expenditures for mothers of moderate or late preterm infants in a level II NICU: Comparison of Alberta Family Integrated Care versus standard care
Source: PEC Innov. 2024 Dec 20;6:100365. doi: 10.1016/j.pecinn.2024.100365 (PMC11732068; doi:10.1016/j.pecinn.2024.100365)
Supplement: Supplementary file 2 — Consumer Price Index adjustment factors used in the analysis [file mmc2.docx]

**Supplementary File 2. Consumer Price Index adjustment factors used in the analysis**

| **Year** | **Adjustment factor*** |
| --- | --- |
| 2015 | 1.272 |
| 2016 | 1.253 |
| 2017 | 1.236 |
| 2018 | 1.207 |

*Direct, non-medical out of pocket expenditures reported in this manuscript were all adjusted to 2024. Adjustment factors used in the analysis were based on the Bank of Canada Consumer Price Index data (<https://www.bankofcanada.ca/rates/related/inflation-calculator/>).
