# Supplementary material for: Direct, non-medical out-of-pocket expenditures for mothers of moderate or late preterm infants in a level II NICU: Comparison of Alberta Family Integrated Care versus standard care
Source: PEC Innov. 2024 Dec 20;6:100365. doi: 10.1016/j.pecinn.2024.100365 (PMC11732068; doi:10.1016/j.pecinn.2024.100365)
Supplement: Supplementary file 3 — Additional analysis of direct non-medical out-of-pocket expenditures [file mmc3.docx]

**Supplementary File 3. Additional analysis of direct non-medical out-of-pocket expenditures**

The manuscript includes the results of the demographic and direct non-medical out-of-pocket expenditure (OOPE) analysis for cases where the proportion of days with *OOPE data* reported was greater than 50% of the infant’s length of stay (LOS). We reported these data in the manuscript to retain the maximum number of cases for analysis while limiting the number of cases with large amounts of potentially missing OOPE data.

Despite encouraging participants to record OOPE daily, even if those values were null, it is difficult to discern if the OOPE missing data was truly missing or was due to zero costs. As such, we have included this supplementary file that contains additional analysis of the parent-reported direct non-medical OOPE.

Tables 1 and 2 in this file report the results of the demographic and direct non-medical OOPE analysis for all cases with *any* parent journal data.

Tables 3 and 4 in this file report the results of the demographic and direct non-medical OOPE analysis for cases where the proportion of days with *any* parent journal data (OOPE or qualitative notes) was greater than 50% of the infant’s LOS.

Except for differences in spending on food, the results of the direct non-medical OOPE analysis are similar between the analyses in this supplement and the analysis in the manuscript. We speculate participants who reported OOPE for over 50% of their infants LOS may have been more accurate, committed, and complete in their reporting of OOPE in data collection forms and this inherent difference may explain the difference in significant findings for the category of food between these supplementary analyses and the manuscript analysis. However, we do have data to confirm this speculation.

**Analysis of All Cases**

- The mean age of mothers in the Alberta FICare™ group (*n* = 247) was 31 (*SD* = 5.59) years old; the mean age of mothers in the SC group (*n* = 145) was 32 (*SD* = 5.20) years old, *t*(390) = 1.642, *p* = 0.101.
- Infants in the Alberta FICare™ group (*n* = 249) had a mean LOS of 18.30 (*SD* = 8.61) days; infants in the SC group (*n* = 151) had a mean LOS of 19.72 (*SD* = 8.02) days, *t*(398) = 1.645, *p* = 0.101.

**Table 1.**

Maternal and infant characteristics by group.

| Maternal characteristics | Alberta FICare^™^  (*n* = 249) | | Standard care  (*n =* 151) | |  |
| --- | --- | --- | --- | --- | --- |
|  | *n* | Frequency (%) | *n* | Frequency (%) | *p* |
| Partnered^a^ | 248 | 237 (95.6) | 148 | 142 (95.9) | .86 |
| Education | 247 |  | 148 |  | .18 |
| High school diploma or less |  | 47 (19.0) |  | 18 (12.2) |  |
| Certificate/diploma |  | 65 (26.3) |  | 39 (26.4) |  |
| College/university degree |  | 135 (54.7) |  | 91 (61.5) |  |
| Employment status | 246 |  | 148 |  | .46 |
| Employed (Full time or part time) |  | 74 (30.1) |  | 37 (25.0) |  |
| Maternity leave^b^ |  | 114 (46.3) |  | 79 (53.4) |  |
| Not employed and not seeking employment |  | 33 (13.4) |  | 21 (14.2) |  |
| Other^c^ |  | 25 (10.2) |  | 11 (7.4) |  |
| Annual family income | 208 |  | 130 |  | .60 |
| < $40,000 |  | 12 (5.8) |  | 7 (5.4) |  |
| $40,000 to $79,999 |  | 43 (20.7) |  | 33 (25.4) |  |
| > $80,0000 |  | 153 (73.6) |  | 90 (69.2) |  |
| Ethnicity | 246 |  | 147 |  | .20 |
| Asian (South, East, Southeast) |  | 27 (11.0) |  | 25 (17.0) |  |
| Black |  | 6 (2.4) |  | 6 (4.1) |  |
| Other^d^ |  | 23 (9.3) |  | 16 (10.9) |  |
| White |  | 190 (77.2) |  | 100 (68.0) |  |
| Born in Canada | 245 | 200 (81.6) | 147 | 107 (72.8) | .08 |
| English as primary language | 245 | 227 (92.7) | 147 | 127 (86.4) | .04 |
| Primiparous | 248 | 139 (56.0) | 150 | 81 (54.0) | .69 |
| Enrolled at urban hospital | 249 | 192 (77.1) | 151 | 132 (87.4) | .01 |
| **Infant characteristics** |  |  |  |  |  |
| Singleton | 249 | 213 (85.5) | 151 | 120 (79.5) | .12 |
| Gestational age (weeks) | 249 |  | 151 |  | .45 |
| 32 weeks |  | 48 (19.3) |  | 23 (15.2) |  |
| 33 weeks |  | 74 (29.7) |  | 42 (27.8) |  |
| 34 weeks |  | 127 (51.0) |  | 86 (57.0) |  |

*Note*. Sample sizes vary due to missing values. ^a^Partnered includes married, common-law, or live-in partner. Not partnered includes single or separated. ^b^ Mother had formalized Canadian maternal leave benefits at the time of demographic survey completion. ^c^Other includes student, not employed but seeking employment, disability or medical leave, and other (e.g. illness, unpaid leave, laid off, contract employment, self-employed). ^d^Includes Indigenous (e.g., First Nations, Inuit, Metis), Latin American, Middle Eastern, and Other/Mixed.

**Table 2.**

Comparison of direct, non-medical out-of-pocket expenditures between Alberta FICare^™^ and standard care groups.

| Expenditure | Alberta FICare^™^  (*n* = 249) | | Standard care  (*n =* 151) | |  |  |
| --- | --- | --- | --- | --- | --- | --- |
|  | Median (IQR) | Minimum, Maximum | Median (IQR) | Minimum, Maximum | *U* | *p* |
| Total | 557 (1261) | 0, 8188 | 652 (981) | 25, 6556 | 16916.50 | .093 |
| Parking^a^ | 0 (0) | 0, 0 | 113 (91) | 0, 515 | 1992.00 | <.001 |
| Food | 241 (396) | 0, 3053 | 200 (249) | 0, 2367 | 19519.00 | .521 |
| Lodging | 0 (15) | 0, 3121 | 0 (0) | 0, 677 | 21805.50 | <.001 |
| Childcare^b^ | 0 (0) | 0, 2494 | 0 (0) | 0, 1485 | 3846.00 | .649 |
| Household Support | 0 (0) | 0, 1026 | 0 (0) | 0, 1378 | 18902.00 | .866 |
| Family Travel | 0 (198) | 0, 5012 | 0 (157) | 0, 4552 | 19446.00 | .510 |
| Miscellaneous | 0 (148) | 0, 3590 | 15 (175) | 0, 1895 | 17939.00 | .411 |

*Note.* Expenditure values reported in 2024 Canadian dollars (CAD) and rounded to the nearest whole number.

IQR = Interquartile range (IQR = Q3-Q1).

^a^ Mothers in the Alberta FICare™ group were assumed to have zero parking costs as they received a parking pass.

^b^Childcare compared only for multiparous mothers (Alberta FICare™, *n* = 109; standard care, *n* = 69).

**Analysis of cases where the proportion of days with *any* parent journal data was greater than 50% of the infant’s length of stay**

- The mean age of mothers in the Alberta FICare™ group (*n* = 239) was 31 (*SD* = 5.58) years old; the mean age of mothers in the SC group (*n* = 139) was 32 (*SD* = 5.09) years old, *t*(376) = 1.711, *p* = 0.088.
- Infants in the Alberta FICare™ group (*n* = 241) had a mean LOS of 17.99 (*SD* = 8.27) days; infants in the SC group (*n* = 145) had a mean LOS of 19.51 (*SD* = 7.88) days, *t*(384) = 1.784, *p* = 0.075.

**Table 3.**

Maternal and infant characteristics by group.

| Maternal characteristics | Alberta FICare^™^  (*n* = 241) | | Standard care  (*n =* 14*5*) | |  |
| --- | --- | --- | --- | --- | --- |
|  | *n* | Frequency (%) | *n* | Frequency (%) | *p* |
| Partnered^a^ | 240 | 229 (95.4) | 142 | 136 (95.8) | .87 |
| Education | 239 |  | 142 |  | .19 |
| High school diploma or less |  | 45 (18.8) |  | 17 (12.0) |  |
| Certificate/diploma |  | 63 (26.4) |  | 37 (26.1) |  |
| College/university degree |  | 131 (54.8) |  | 88 (62.0) |  |
| Employment status | 238 |  | 142 |  | .44 |
| Employed (Full time or part time) |  | 72 (30.3) |  | 35 (24.6) |  |
| Maternity leave^b^ |  | 110 (46.2) |  | 77 (54.2) |  |
| Not employed and not seeking employment |  | 32 (13.4) |  | 19 (13.4) |  |
| Other^c^ |  | 24 (10.1) |  | 11 (7.7) |  |
| Annual family income | 202 |  | 125 |  | .79 |
| < $40,000 |  | 10 (5.0) |  | 6 (4.8) |  |
| $40,000 to $79,999 |  | 42 (20.8) |  | 30 (24.0) |  |
| > $80,0000 |  | 150 (74.3) |  | 89 (71.2) |  |
| Ethnicity | 238 |  | 141 |  | .17 |
| Asian (South, East, Southeast) |  | 24 (10.1) |  | 23 (16.3) |  |
| Black |  | 6 (2.5) |  | 6 (4.3) |  |
| Other^d^ |  | 23 (9.7) |  | 16 (11.3) |  |
| White |  | 185 (77.7) |  | 96 (68.1) |  |
| Born in Canada | 237 | 195 (82.3) | 141 | 103 (73.0) | .03 |
| English as primary language | 238 | 220 (92.4) | 141 | 122 (86.5) | .06 |
| Primiparous | 240 | 133 (55.4) | 144 | 75 (52.1) | .53 |
| Enrolled at urban hospital | 241 | 185 (76.8) | 145 | 126 (86.9) | .02 |
| **Infant characteristics** |  |  |  |  |  |
| Singleton | 241 | 205 (85.1) | 145 | 115 (79.3) | .15 |
| Gestational age (weeks) | 241 |  | 145 |  | .43 |
| 32 weeks |  | 46 (19.1) |  | 22 (15.2) |  |
| 33 weeks |  | 71 (29.5) |  | 39 (26.9) |  |
| 34 weeks |  | 124 (51.5) |  | 84 (57.9) |  |

*Note*. Sample sizes vary due to missing values. ^a^Partnered includes married, common-law, or live-in partner. Not partnered includes single or separated. ^b^ Mother had formalized Canadian maternal leave benefits at the time of demographic survey completion. ^c^Other includes student, not employed but seeking employment, disability or medical leave, and other (e.g. illness, unpaid leave, laid off, contract employment, self-employed). ^d^Includes Indigenous (e.g., First Nations, Inuit, Metis), Latin American, Middle Eastern, and Other/Mixed.

**Table 4.**

Comparison of direct, non-medical out-of-pocket expenditures between Alberta FICare^™^ and standard care groups.

| Expenditure | Alberta FICare^™^  (*n* = 241) | | Standard care  (*n =* 14*5*) | |  |  |
| --- | --- | --- | --- | --- | --- | --- |
|  | Median (IQR) | Minimum, Maximum | Median (IQR) | Minimum, Maximum | *U* | *p* |
| Total | 557 (1206) | 0, 8188 | 724 (1073) | 25, 6556 | 15442.00 | .056 |
| Parking^a^ | 0 (0) | 0, 0 | 115 (81) | 0, 515 | 1687.00 | <.001 |
| Food | 243 (400) | 0, 3053 | 201 (249) | 0, 2367 | 18106.00 | .551 |
| Lodging | 0 (0) | 0, 3121 | 0 (0) | 0, 677 | 20122.50 | <.001 |
| Childcare^b^ | 0 (0) | 0, 2494 | 0 (0) | 0, 1485 | 3784.00 | .619 |
| Household Support | 0 (0) | 0, 1026 | 0 (0) | 0, 1378 | 17601.50 | .822 |
| Family Travel | 0 (185) | 0, 5012 | 0 (163) | 0, 4552 | 18079.50 | .513 |
| Miscellaneous | 0 (152) | 0, 3590 | 10 (175) | 0, 1895 | 16725.50 | .450 |

*Note.* Expenditure values reported in 2024 Canadian dollars (CAD) and rounded to the nearest whole number.

IQR = Interquartile range (IQR = Q3-Q1).

^a^ Mothers in the Alberta FICare™ group were assumed to have zero parking costs as they received a parking pass.

^b^Childcare compared only for multiparous mothers (Alberta FICare™, *n* = 107; standard care, *n* =69).
